# 3D Printing of Polymer Waste for Improving People’s Awareness about Marine Litter

**DOI:** 10.3390/polym12081738

**Published:** 2020-08-04

**Authors:** Francesca Ferrari, Carola Esposito Corcione, Francesco Montagna, Alfonso Maffezzoli

**Affiliations:** Department of Engineering for Innovation, University of Salento, Via Arnesano, 73100 Lecce, Italy; francesca.ferrari@unisalento.it (F.F.); francesco.montagna@unisalento.it (F.M.); alfonso.maffezzoli@unisalento.it (A.M.)

**Keywords:** PET, recycling, 3D printing

## Abstract

This work is aimed at proposing demonstrative actions devoted to show reprocessing and recyclability of PET originating from bottles collected from the seaside, in order to increase the consumer awareness on the importance of recycling plastics. To this purpose, collected bottles were washed, cut, grinded, extruded in the form of a thin wire adopting different cooling rates, which leads to a modulation of the crystallinity content. Once having optimized the processing parameters, the extruded wire was used to produce 3D printed samples through the fused deposition modelling (FDM). The changes in the crystalline structure due to the different processing conditions were assessed by DSC and XRD analyses, while rheological tests were performed in order to evaluate any modification in the viscosity of PET after repeated processing cycles. The reduction in thermal stability was confirmed by TGA analysis, which showed a progressive decrease in the degradation temperature as processing cycles increased. Finally, tensile tests highlighted the difference in the mechanical response due to the predominance of the crystalline or amorphous phase in the tested sample. In particular, a good mechanical behavior was found for the 3D-printed samples.

## 1. Introduction

Nowadays, huge population growth and the associated use of polymers for disposable objects and packaging cause uncontrolled waste production, with consequent serious problems due to its management and disposal [1]. In particular, a great challenge for waste management is linked to the uncontrolled waste stream coming from different sources [2,3].

Plastics represent a considerable fraction of municipal solid waste, whose worldly production passed from 1.3 billion tons in 1990 to 3.81 billions tons after 25 years [4,5]. Among these, the highest contribution is given by thermoplastic polymers, whose consumption, about 80% of all synthetic polymers, is mainly attributable to packaging and containers, as well as the production of textile fibers [6]. Plastics play a key role because of their extensive usage, their short service life, but their long (bio)degradation time, thus leading, nowadays, to the development of alternative strategies, such as recycling or production of biomaterials coming from renewable sources [7]. The daily use of plastics involves several applications, such as greenhouses, mulches, fishing nets, coating and wiring, packaging films, trays and bottles, covers, bags, and containers [8,9]. The most common ways to manage plastic wastes are incineration and landfill, both with a potential negative environmental impact [10]. In particular, incineration of plastics involves the production of hazardous gases and the release of dangerous compounds, such as sulphur oxides, ashes, dioxin, and other toxic compounds [11]. On the other hand, the disposal in landfills should be avoided, since plastics are predominantly not biodegradable and thus require very long storage times [12]. Also, a major concern due to storage in landfills consists in the readily availability of the plastics in the environment. Mismanaged plastic Dwaste, and in particular of polyethylene containers and poly(ethylene terephthalate) bottles, the most common polymers found in urban waste, leads to a huge amount of surface water and seabed marine litter [13]. A particular class of litter found in the oceans, including the Antarctic one [14], is so-called microplastics, whose amount has been increasing in the sea for at least four decades [15,16]. Microplastics derive from plastic pollution of seaside and beaches, and consist of micron-size plastics originating from fragmentation phenomena or originating from powders used for instance in cosmetics [17]. The size of microplastics varies from a few µm to 500 µm, whereas bigger particles, such as pellets, are called mesoplastics [18]; both kind of wastes, which belong to persistent organic pollutants (POPs), are eaten by marine species and, as a consequence, can reach the marine food web. In contrast to bigger plastic wastes, microplastics on the seaside, seabed, or surface water often mixed with sand, are difficult to be collected, and currently, there is not a standard procedure for the determination of their amount. Moreover, solar light degradation of marine microplastics can induce the production of nanoplastics in marine water. Gigault et al. [19] discovered for the first time the presence of nanoplastics in water caused by UV exposure of microplastics. In particular, authors attributed the formation of nanoplastics to an advanced state of oxidation of plastics found on the seaside. Therefore, the formation of small nano-plastics (i.e., <100 nm) was induced by previous degradation in natural systems before sunlight exposure.

The arising environmental hazards caused by common waste management methods, the decreased space of landfills and the exponential increase in marine pollution require alternative solutions for plastic waste disposal [20]. As a consequence, recycling of plastic wastes is nowadays rapidly growing as it is assumed to be the best approach for the reduction of environmental pollution caused by landfill. Moreover, recycling of plastics allows lower energy and oil-based raw materials consumption [21].

As mentioned before, poly(ethylene terephthalate) is one of the most common plastics found in the seaside, mainly as closed bottles of beverages. Commonly coded as PET or PETE, it is a thermoplastic polymer of the polyesters family employed for the production of containers for drinks, food, and for synthetic fibers manufacturing. Its widespread use is mainly due to a mix of good properties (high strength, stiffness, low density, good creep behavior, and high chemical resistance) and low cost [22].

The degree of crystallinity of PET, which can be both amorphous or semicrystalline, depends on the processing and the thermal conditions, such as different cooling rates and stretching conditions. All the physical and mechanical properties of PET are strongly affected by the crystallinity. Therefore, the recycling process of the post-consume PET [23,24] is not so simple as can be expected, since it is a cross-disciplinary practice that must take into account polymer chemistry, physics, processing, and manufacturing engineering [25].

Most of the consumers of plastic products are not aware of how they can be recycled and which objects can be produced from recycled polymers. This work has been developed within the European project called RE.CO.RD (REcycling strategies for the COastal sustainable waste management towards R&D Innovation) which includes some activities related to increasing the awareness of people to the relevance of recovering and recycling the marine litter originated by plastics. To this aim, demonstration of the production of 3D-printed PET objects starting from bottles collected from the seaside was performed in public events. This work was aimed to set up a procedure to develop demonstrative actions, for instance, in schools or on beaches, devoted to increasing people awareness about the importance of recovering and recycling the marine plastic waste, and in general, following the same methodology, the plastic waste collected from other sources. Thanks to the small size of 3D printers, a true fabrication procedure with recycled plastic can be easily performed, producing a large variety of geometries in an easy way and in a short time [26]. 

This study reports how PET bottles collected at the sea shore can be used in 3D printing: after being collected and washed they are cut, grinded, and extruded to obtain a wire. Afterwards, the extruded wires are used to produce 3D-printed samples through fused deposition modelling (FDM), one of the most widely used additive manufacturing techniques for rapid prototyping of polymer and composites components, being characterized by simplicity, high speed, and low cost. Recent studies showed the possibility to use recycled PET for 3D print, whose properties were found better compared to virgin PET. The recycling of PET showed, in fact, a potential as an alternative filament for 3D printing [27,28]. 

## 2. Materials and Methods

### 2.1. Production of the Wire and 3D Print

The first step of PET recycling involved the collection of the bottles from the Italian Ionian coast on the Mediterranean Sea. Once collected and washed in water, with repeated cycles until a complete removal of dust, PET bottles were cut and milled using a RETSCH SM2000 mill (Düsseldorf, Germany). The obtained flakes were desiccated at 70 °C overnight and then extruded by a Haake QC Rheomex single screw extruder (Waltham, MA, USA) In addition, 60 mm × 12 mm × 0.1 mm stripes were cut from the bottles and desiccated in order to obtain samples for tensile tests.

The extrusion process was run at a barrel temperature profile of 240–270–280–220 °C with a screw speed of 10 rpm and an average mass flow rate of 1 kg/h. Different cooling systems were applied at die exit, in order to control the effect of the cooling rate on the crystallinity of the wire. In particular, a faster cooling was obtained by using compressed air, instead of natural convection cooling to room temperature. PET wires, obtained both with slow and rapid cooling, were used for tensile tests. In particular, tested samples were 70 mm long, with an average diameter of 0.6 mm.

**Figure 1:**
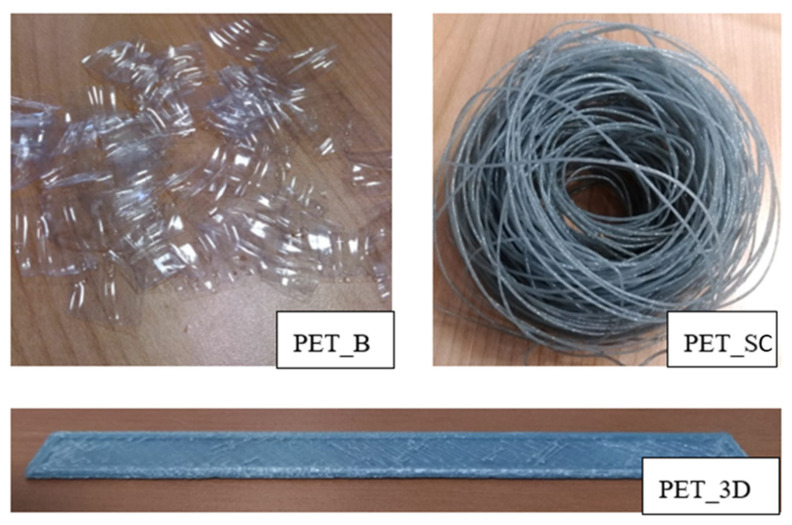
Different processing steps from PET flakes to PET wire and PET 3D sample.

3DPRN LAB 3D printer (TIPS srl, Città Sant’Angelo, Italy), operating with the FDM (Fused Deposition Modeling) approach, was adopted to build the samples, layer-by-layer, by extruding the PET wire. All the samples were printed using a nozzle size of 0.4 mm, a layer height of 0.2 mm, a speed extrusion of 50 mm/s, and setting the temperature at 250 °C. 3D-printed samples, 60 mm long, 13.2 mm wide, and with two average thicknesses (0.7 mm for tensile tests samples and 3 mm for MicroCT analysis), were obtained. In order to consider the difference in thickness due to the overlap of the different layers during the print, the average thickness of each sample was calculated by dividing its weight for the length and the width. Figure 1 shows the different processing steps, starting from PET bottle flakes to the 3D printed PET specimen for mechanical testing. All the produced samples were tested and compared with those obtained using commercial PET pellets, in order to compare the effect of aging on thermal, morphological, and mechanical properties.

All the tested samples were labelled as follows:

- PET pellets: PET_P.
- PET flakes from bottles: PET_B
- extruded wire, rapid cooling: PET_RC
- extruded wire, slow cooling: PET_SC
- 3D printed sample from extruded wire, rapid cooling: PET_3D

### 2.2. Analysis of the Degree of Crystallinity

XRD analysis (Rigaku, Tokyo, Japan) was carried out with CuKα radiation (λ = 1.5418 Å) in the step scanning mode recorded in the 2θ range of 10°–40°, with a step size of 0.02° and step duration of 0.5 s.

DSC analysis was performed on a Mettler Toledo 822 (Mettler Toledo, Greifensee, Switzerland) instrument under a nitrogen flux of 60 mL/min, applying a heating scan between 20 and 300 °C at 10 °C/min.

### 2.3. Rheological and Thermogravimetric Analysis 

Rheological analyses were carried out on a Rheometrics Ares rheometer (TA Instruments, New Castle, DE, USA). Steady rate tests were performed at 260 °C, with a cone and plate geometry, varying the shear rate from 0.05 to 10 s^−1^. 

A simultaneous thermal analyzer Netzsch STA 409 (Netzsch, Selb, Germany) was used for thermogravimetric (TGA) characterization, heating all the samples from room temperature to 700 °C at 10 °C/min in air. 

### 2.4. Mechanical Characterization

Tensile tests were performed on PET bottles, extruded wires [29,30], and 3D printed samples (60 mm long, 13.2 mm wide, and 0.7 mm thick) [31] using a Lloyd LR50K dynamometer (Lloyd Instruments Ltd, Bognor Regis, UK). Six replicates for each measurement were performed, in order to obtain statistically relevant results. As reported above, tensile tests were directly carried out on extruded wires with a length of 70 mm and an average diameter of 0.6 mm, in order to evaluate the effect of different cooling rates on the mechanical response of PET. 

### 2.5. Analysis of the Porosity Distribution

**Figure 2:**
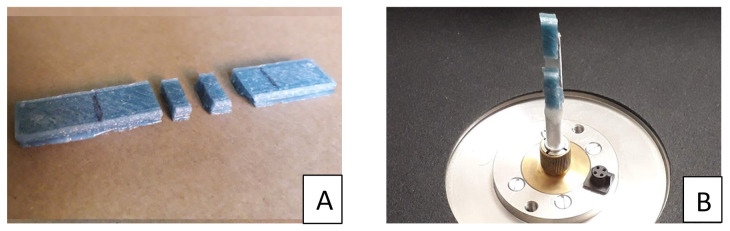
Cut samples for MICRO CT analysis (**A**) and housing in the tomograph holder (**B**).

The analysis of the porosity was performed by an X-ray microtomography system (Skyscan 1072) on the central area of the 3D printed specimen, as shown in Figure 2A. A sample of 3 × 13.2 × 3 mm^3^ was loaded on the sample holder as showed in Figure 2B. 

The morphology of the 3D printed sample was characterized by an scanning electronmicroscope (SEM, Zeiss evo 40, Oberkochen, Germany) equipped with an energy dispersive X-ray (EDX) spectroscopysystem (Bruker x flash detector 5010, Billerica, MA, USA). The pores distribution and dispersion on the cross-sectional surface was evaluated.

## 3. Results and Discussion

### 3.1. Analysis of the Degree of Crystallinity

**Figure 3:**
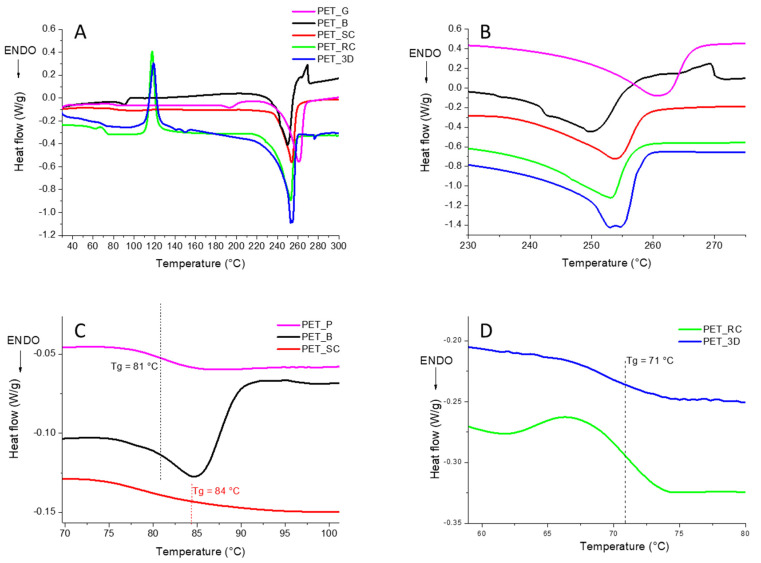
DSC analysis of PET samples (**A**). Temperature interval of melting (**B**) and glass transition (**C**,**D**).

DSC analysis was carried out in order to check the difference in the crystalline structure of the tested samples. The value of the *T*_g_ was calculated by identifying the point corresponding to the presence of an inflection (inflection point method). Thus, the *T*_g_ coincides with the point at which the second derivative is zero. As shown in Figure 3C,D, a lower degree of crystallinity involved a decrease in glass transition temperature, which shifted from 81 °C (PET_B and PET_P) and 84 °C (PET_SC) to 71 °C for PET_RC and PET_3D samples. Also, as a consequence of the cooling history resulting from their peculiar fabrication processes, PET_B samples are characterized by relaxation peaks at *T*_g_.

The glass transition behavior of semi-crystalline polymers is correlated to molecular weight, amount of crystalline phase, and crystal morphology [32,33]. In particular, the mobility of the amorphous phase for semi-crystalline PET can be affected by the proximity of crystallites. Alves et al. [34] reported that PET shows two amorphous phases, and as a consequence, two different values of glass transition, based on the closeness of the amorphous phase to the crystalline region: a lower glass transition is found when the amorphous phase is far from the spherulites, whereas polymer chains near to the crystallites are characterized by higher glass transition temperature. As a consequence, a higher degree of crystallinity corresponds to a higher and broader glass transition temperature [35]; in particular, it has been reported a *T*_g_ of 67 °C for amorphous PET and 81 °C for semi-crystalline PET [18].

Besides their influence on the glass transition temperature, different cooling conditions strongly affect both the crystallization and the melting of the polymer. Crystallinity in PET can be induced by thermal crystallization and/or by stress- or strain-induced crystallization. The classic processes for bottles production are based on blowing amorphous preforms obtained by injection molding. During the process, crystallization is associated with molecular orientation induced by radial and circumferential strain, which involves a sub micrometric distribution of both the amorphous and the crystalline phase. On the other hand, no orientations or stretching were applied after the extrusion of the wire obtained from PET bottles, therefore it can be assumed that thermally-induced crystallization of PET_SC samples occurred under slow cooling, leading to the formation of an un-oriented spherulitic crystal structure [36]. This crystalline morphology is not found in commercial products since it is responsible of very high brittleness and loss of transparency. 

The increase in cooling rate obtained by using compressed air involved a modification of the crystalline fraction. Referring to Figure 3A, The DSC curve of compressed air cooling samples showed the presence of the cold crystallization peak at about 110 °C. The presence of this peak even in 3D printed samples indicates that the cooling rates of FDM process are high enough to quench the polymer leading to a metastable amorphous structure. On the other hand, DSC curves of PET_SC wires did not show the presence of cold crystallization, thus indicating that the slower cooling involved the full development of the crystalline phase.

An initial estimation of the degree of crystallinity *x_cDSC_* can be obtained from Figure 3A as:

(1) $$x_{cDSC}=\frac{H_{m}-H_{cc}}{H_{m}}$$

where *H_cc_* and *H_m_* are the cold crystallization and melting enthalpies measured during the DSC scan and reported in Table 1. The melting enthalpy is the sum of the melt crystallization and the cold crystallization enthalpy. This does not occur often since the crystals are modified during heating above *T*_g_. During the heating process above the cold crystallization temperature range, lamellar thickness increases by multiple melting recrystallization processes characterized by a zero net enthalpy and therefore not detected in a DSC scan. Therefore, the absolute value of melting enthalpy is usually higher than absolute value of cold crystallization enthalpy being the former associated to higher lamellar thicknesses than the latter. 

Alternatively, the degree of crystallinity of PET can be obtained by dividing the difference (*H_m_* − *H_cc_*) by the theoretical crystallization enthalpy of a completely crystalline PET (*H_m_*_100_), which is 140 J/g [37]:

(2) $$x_{cT}=\frac{H_{m}-H_{cc}}{H_{m100}}$$

The results, reported in Table 1, indicate higher crystalline fractions for PET samples before the extrusion, both for pellets and flakes. The extrusion of PET involved, in any case, a reduction of the crystalline fraction. The main reason for a decreased crystallinity can be found in the cooling conditions after processing, leading to a lower lamellar thickness, i.e., the “crystallizable fraction” of the polymer is always the same but is distributed between the lamellar surface and the bulk in a different ratio depending on the thickness of the lamellae [38]. This is confirmed by the melting temperature (Figure 3B), directly proportional to lamellar thickness, which, as reported in Table 1, is higher for PET pellets (PET_P).

Similarly, natural air cooling led to a higher crystalline degree, compared to compressed air cooling, leading to a partial quenching. This is mainly due to the faster cooling obtained with compressed air; in particular, although the minimum cooling rate required to produce non-crystalline PET is dependent on molecular weight, an increase in the heating rate always leads to a decrease in the crystalline fraction. Moreover, PET_3D samples showed the presence of a cold crystallization peak, though higher *x*_c_ than PET_RC wires, indicating a partial quenching. Even if 3D printing is apparently performed in slow cooling conditions, i.e., in natural convection conditions, PET melt is cooled faster by the conductive heat transfer originated by the contact of each layer during deposition with the solid and cool substrate, thus inducing a decrease in the crystalline fraction. 

**Figure 4:**
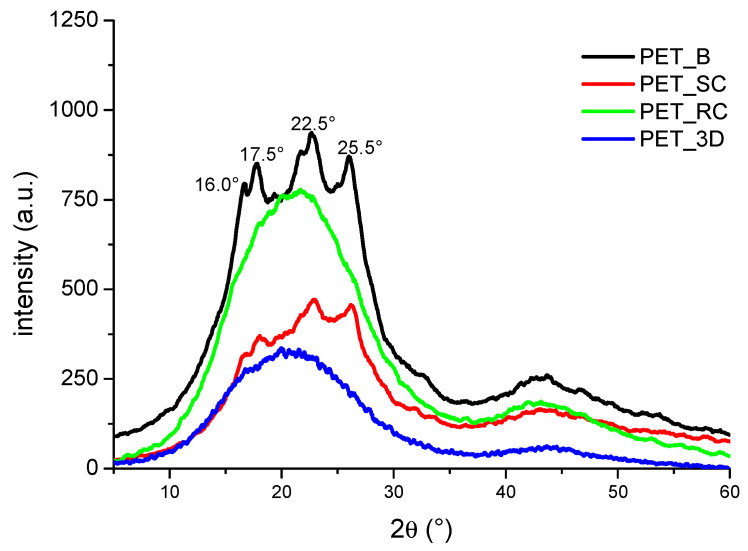
XRD of PET samples.

A more accurate measurement of the crystallinity of extruded and 3D PET samples can be obtained by XRD analyses. In this case, the sample is analyzed at a room temperature, while the use of DSC affects the crystal morphology during heating between *T*_g_ and *T*_m_, where crystal perfection and multiple melting and re-crystallization phenomena occur. The molecular orientation caused by the blowing injection process adopted for PET bottles induced bi-orientation of semi-crystalline PET crystals on the stretching and circumferential direction. Therefore, PET_B samples show the presence of peaks at 2θ = 16.0°, 17.5°, 22.5°, 25.5°, which correspond, respectively, to crystalline planes with Miller index of (010), (010), (110), and (100) [39]. Orientation was lost after PET extrusion and PET_SC samples showed a random distribution of the planes with weaker intensity, partially covered by the scattering of the amorphous phase. Finally, the diffraction pattern of PET_RC and PET_3D samples indicates a predominance of the amorphous phase signal. The calculation of the degree of crystallinity *x_cXRD_* was carried out by using the following equation [40]:

(3) $$x_{cXRD}=\frac{Q_{st}-Q_{am}}{Q_{st}}\times 100$$

where *Q_st_* and *Q_am_* are the areas calculated under the X-ray curves from the semi-crystalline and amorphous samples, respectively. The baseline for the semi-crystalline area was traced by taking into account previous studies performed by D. E. Bosley [41]. The results, reported in Figure 4 and in Table 1, confirm the progressive decrease in crystalline fraction from PET_B to PET_SC. PET_RC and PET_3D can be considered almost completely amorphous.

### 3.2. Rheological and Thermogravimetric Analysis

Rheological analyses were performed on PET samples in order to evaluate the change in viscosity of the material after being subjected to repeated processing cycles. All the samples showed a moderate pseudo-plastic behavior, characterized by a slight viscosity decrease with increasing shear rate. Therefore, by considering a power law correlation between the viscosity and the shear rate:

(4) $$\eta =\eta_{0}{\dot{\gamma }}^{n-1}$$

**Figure 5:**
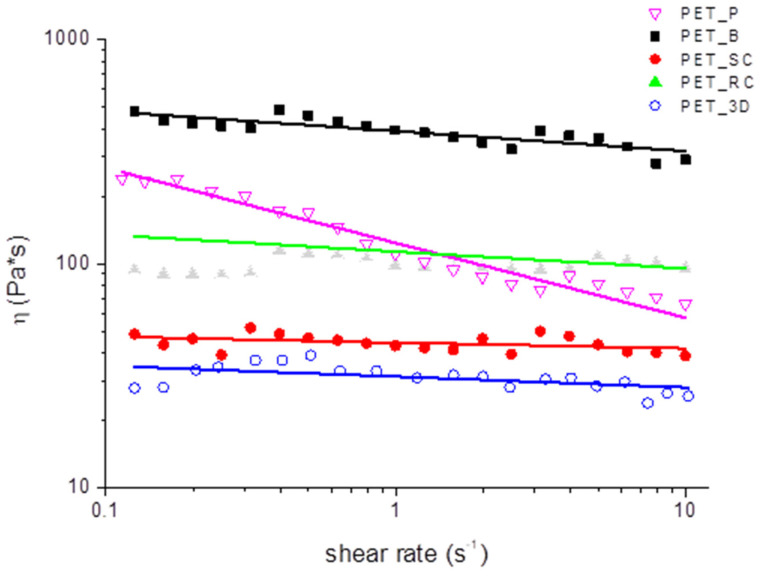
Viscosity curves and regression line on PET samples at 260 °C.

The *n* index was calculated by fitting the curves in Figure 5, in the shear rate range 0.5–10 s^−1^, and reported in Table 2. The n index of all processed PET samples, beside PET_P, was similar and above 0.9. On the other hand, PET_P, i.e., unprocessed pellets, showed a much lower value of n. Since the non-Newtonian behavior of a polymer is dependent on its average molecular weight (*M*_w_), with *n* increasing with decreasing *M*_w_ [42,43], it can be reasonably assumed that each processing step reduces *M*_w_ and this is more evident for PET_3D, which underwent two processing cycles, and PET_SC, which was kept at a high temperature for a longer time than the other samples as a consequence of the slow cooling.

In particular, a *n* index very close to 1, as for PET_SC and PET_3D, indicates an almost Newtonian behavior, which indeed can be attributed to reduced average molecular weight. If the average molecular weight of the polymer is much reduced, then the rheological behavior can be transformed from non-Newtonian (which is dominant in pseudo-plastic material) to Newtonian. The reduction of molecular weight, which occurs after extrusion, can be ascribed to hydrolysis of ester bonds, basically due to the high temperature the material is exposed during processing, occurring even in the presence of a very small amount of absorbed moisture of the material.

**Figure 6:**
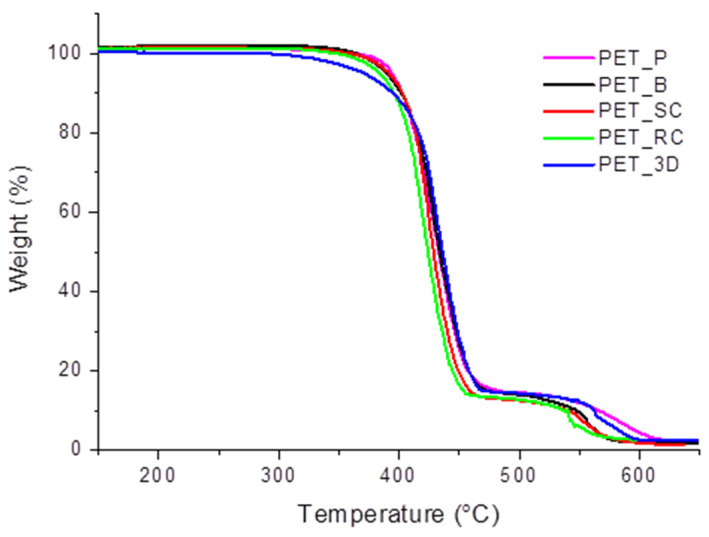
TGA curves on PET samples.

A reduction in thermal stability was confirmed by TGA analysis. The results, shown in Figure 6 and reported in Table 3, indicate a progressive decrease of the onset of degradation (*T*_onset_, calculated as the point of intersection of the starting-mass baseline and the tangent to the TGA curve at the point of maximum gradient) with increasing processing steps. In particular, as reported in Figure 6, the lowering in degradation temperature is proportional to the number of processing cycles: a decrease of about 10 °C was found between pellets and bottle flakes. Wire fabrication by extrusion involved a further reduction of *T*_onset_ of 20 °C compared to PET_B. Finally, the highest decline in thermal stability was detected for PET_3D samples, which are characterized by a *T*_onset_ lower than 40 °C compared to the that of extruded wires.

The solid residue was calculated for all the curves in the temperature range of 600–640 °C and reported in Table 3 with its standard deviation. Results show that the residue is about the same for all samples.

### 3.3. Mechanical Characterization

**Figure 7:**
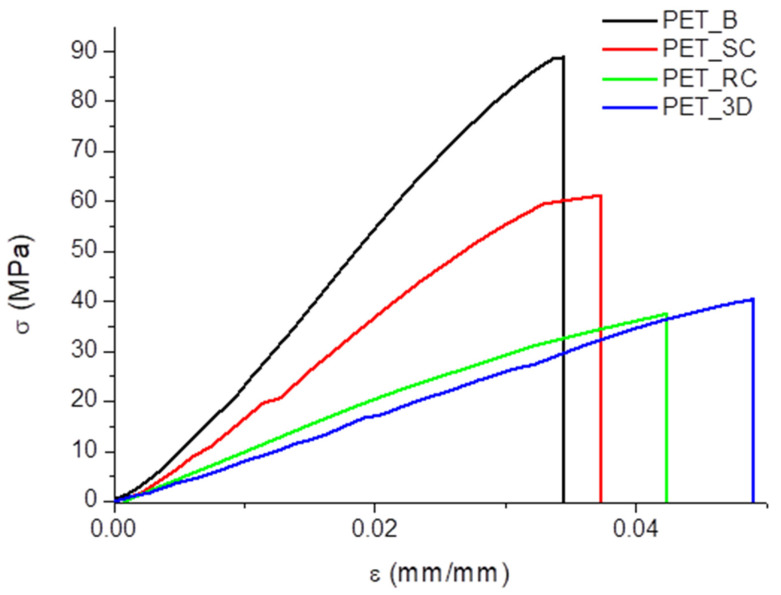
Stress-strain average curves.

Tensile tests were performed on PET samples, subjected to progressive processing cycles, in order to evaluate the effect of recycling on the mechanical response of the material. However, the mechanical properties result from complex counteracting effects due to a reduction of the molecular weight, depending on the number of processing cycles, and the degree of crystallinity, depending either on molecular weight or on processing conditions. For such a reason, an average value of mechanical properties of neat PET, whose strength at break can vary between 37 and 80 MPa [44], was reported in Table 4. The data reported in Figure 7 show that a very limited plastic deformation, if any, is observed for all samples. The increase of modulus and strength and the reduction of strain to break with the degree of crystallinity suggest that this is the most relevant factor acting on mechanical properties. However, PET_B and PET_SC are characterized by a relatively small difference in the degree of crystallinity and in this case the decrease of molecular weight due to an additional processing cycle that can play a role. PET_RC and PET_3D are both characterized by substantially similar behavior also looking at the standard deviation for all mechanical parameters. Finally, it must be highlighted that slow crystallized PET is always too brittle either to make wires for 3D printing, or to make plastic parts for 3D printing. Therefore, the goal of wire extrusion and FDM is to obtain an amorphous polymer characterized by an adequate tenacity, which is much less relevant than the elastic modulus and the strength. As shown in Table 4, mechanical properties of polylactic acid [45], by far the most used polymer for 3D printing by FDM, are comparable to those obtained with recycled PET in this study.

### 3.4. Micro CT Analysis

**Figure 8:**
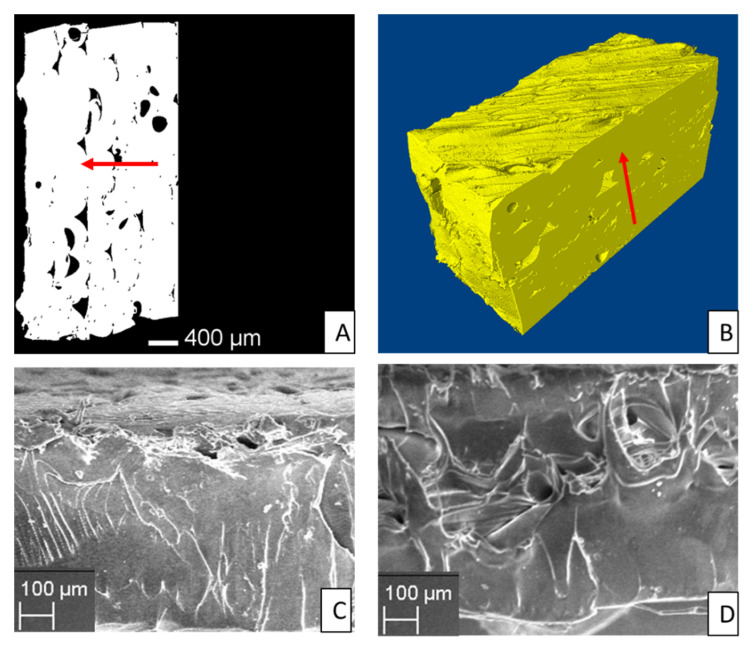
Micro CT analysis; segmented cross section (**A**) and 3D model from CT (**B**). Red arrows indicate the deposition direction. SEM images of the cross section of PET_3D sample (**C**,**D**).

Figure 8A,B shows micro CT analysis of a PET_3D sample obtained using the recycled PET wire (PET_RC). Most of the porosities can be associated to interface among wires, indicating that the extrusion process led to an adequately uniform material. The high temperature required for 3D printing of recycled PET wires can determine non-uniform melting leading to the interlayer porosity reported in Figure 8.

The calculation of the total porosity of the analyzed samples is given by the sum of closed and open pores. The analyzed samples present a relatively high porosity, although the porosity is mostly composed of open pores. The total porosity, mainly given by open one, ranges between 3.5% and 7% This porosity level is comparable with that obtained using standard materials like PLA in 3D printing [47]. The measured porosity provides a further contribution to property loss of 3D printed materials compared to that the bottle grade (Figure 7), besides crystal content and loss of molecular weight, above cited.

The results of micro-CT were confirmed by SEM analysis of the cross section of the PET_3D sample. Figure 8C,D show the presence of small holes distributed on the sample surface; in particular, in accordance with Micro CT analysis, most of porosity is found at the deposition line of the 3D-printed sample.

## 4. Conclusions

Following the research activity carried out in the framework of the RECORD project (REcycling strategies for the COastal sustainable waste management towards R&D Innovation), this work was aimed to increase people’s awareness about the importance of recovering and recycling marine plastic waste. To this purpose, PET bottles, collected from the seaside, were washed, grinded, and extruded with different cooling rates, thus allowing a modulation of the crystalline degree of PET. The produced wire was then employed for the production of 3D-printed PET objects.

The degree of crystallinity of PET was assessed by DSC and XRD analyses, which showed how cooling conditions affected the crystallinity and the lamellar thickness.

The reduction in thermal stability after repeated processing cycles was then studied by rheological and TGA analyses. In particular, the variation of the n index suggests a lowering of the average molecular weight with reprocessing.

Tensile tests, carried out on PET samples after repeated processing cycles, showed a change in the mechanical response of the material; since the mechanical response of the polymer is contemporarily affected by the degree of crystallinity and the decrease in molecular weight, the reduction of tensile strength can also be attributed either to the complex interaction of these variables either to the presence of porosities, mainly in 3D printed samples.

On the other hand, a higher tenacity was detected for samples subjected to rapid cooling. This result suggests that recycled PET, providing that processing conditions lead to an amorphous polymer, can be adopted in 3D printing, contributing to the environmental sustainability of this technology, mainly for its role in increasing consumer awareness about plastics disposal and their recyclability.

## Figures and Tables

**Table 1:** Properties of studied PET samples. *T*_g_ was measured as the inflection point and *T*_m_ at the endothermic peak.

Sample	*T*_g_ (°C)	*T*_m_ (°C)	*H*_C_ (J/g)	*H*_M_ (J/g)	*x*_cDSC_	*x*_cT_	*x*_cXRD_
PET_P	81	261.2	--	50.67	1	0.36	--
PET_B	81	249.8	--	52.82	1	0.38	0.28
PET_SC	84	253.9	--	41.67	1	0.30	0.22
PET_RC	71	252.8	29.92	48.87	0.43	0.13	0
PET_3D	71	253.9	30.75	65.11	0.53	0.24	0

**Table 2:** *n* index of samples obtained by power law.

SAMPLE	*n*
PET_P	0.69
PET_B	0.88
PET_SC	0.97
PET_RC	0.92
PET_3D	0.94

**Table 3:** Tonset and solid residue from TGA curves.

SAMPLE	*T*_onset_ (°C)	*T*_95% weight loss_ (°C)	Solid Residue (%)
PET_P	369.43	596.96	2.34 ± 0.62
PET_B	359.74	564.57	1.92 ± 0.88
PET_SC	353.83	564.52	1.56 ± 0.91
PET_RC	351.56	555.32	2.56 ± 0.47
PET_3D	329.44	584.08	2.67 ± 0.33

**Table 4:** Mechanical properties of PET samples.

SAMPLE	σ (MPa)	ε (%)	*E* (GPa)
NEAT PET [46]	59.62 ± 2.9	5.22 ± 1.60	2.47 ± 1.98
PET_B	89.2 ± 9.16	3.12 ± 0.81	2.71 ± 0.23
PET_SC	63.0 ± 6.79	3.73 ± 0.78	2.19 ± 0.32
PET_RC	39.1 ± 5.21	4.20 ± 0.74	1.14 ± 0.22
PET_3D	40.9 ± 6.18	5.15 ± 0.89	1.01 ± 0.36
PLA [42]	63.5 ± 4.61	4.21 ± 0.44	2.40 ± 0.21
